# Antimicrobial Activity Does Not Predict Cytokine Response to Adrenomedullin or Its Shortened Derivatives

**DOI:** 10.1155/2007/30987

**Published:** 2007-10-10

**Authors:** Quratul Ann Hussain, Barry E. Sheehan, Ian J. McKay, Robert P. Allaker

**Affiliations:** ^1^Institute of Dentistry, Queen Mary, University of London, Newark Street, London E1 2AT, UK; ^2^Department of Asthma, Allergy and Respiratory Science, Kings College London,5th Floor, Thomas Guy House, Guy's Hospital, London SE1 9RT, UK

## Abstract

The aim of this study was to investigate cytokine release from oral keratinocytes and fibroblasts in response to AM and shortened derivatives previously characterised in terms of their antimicrobial activities. Cells were incubated with AM or its fragments (residues 1-12, 1-21, 13-52, 16-21, 16-52, 22-52, 26-52, and 34-52), and culture supernatants collected after 1, 2, 4, 8, and 24 hours. A time-dependant increase in production of interleukin1-α and interleukin 1-β from keratinocytes in response to all peptides was demonstrated. However, exposure to fragments compared to whole AM resulted in reduced production of these cytokines (60% mean reduction at 24 hours, P<.001). No consistent differences were shown between the cytokine response elicited by antimicrobial and nonantimicrobial fragments. The production of interleukin-6 and interleukin-8 did not change significantly with time or peptide used. Fibroblast cells were relatively unresponsive to all treatments. This study demonstrates that antimicrobial activity does not predict cytokine response to adrenomedullin or its shortened derivatives.

## 1. INTRODUCTION

Adrenomedullin (AM) is a 52 amino acid multifunctional peptide [1] produced by a wide variety of tissues and cells. Previous studies have demonstrated that AM has antimicrobial activity against a number of members of the normal skin, oral, respiratory tract, and gut microflora [2]. Carboxy-terminal fragments (residues 13-52, 16-52, 22-52, and 34-52) of AM have been shown to be up to 250-fold more active than the parent molecule in terms of antimicrobial activity. Whereas, AM fragments (1-12, 1-21, 16-21, and 26-52) were found to be inactive [3]. Further support for a role in host defence has been provided by a study to demonstrate that protein and mRNA levels are increased when oral keratinocytes are exposed to whole bacteria and culture supernatants [4].

It is also recognised that AM influences the inflammatory response to infection. The regulation by AM of cytokine production from cultured rat macrophages in response to lipopolysaccharide (LPS) has been previously investigated [5, 6]. AM was shown to significantly reduce LPS-induced TNF-*α* production by macrophages. In contrast, AM up-regulated the production of the cytokine IL-6 in both LPS-stimulated and unstimulated macrophages. Other findings also support the role of AM as a proinflammatory factor, with both migratory inhibitory factor (MIF) and IL-1*β* significantly increased in the presence of AM [6]. These somewhat conflicting results emphasise the complexity of the interactions between AM and the inflammatory system.

Postsecretory processing of the cathelicidin LL-37 peptide has been shown to generate fragments with enhanced antimicrobial activity and a marked decrease in their ability to stimulate IL-8 production from cultured keratinocytes [7]. It is also known that structural modifications of defensins have significant effects on both chemotactic function and antimicrobial activity [8]. Thus it is hypothesised that postsecretory processing of the AM molecule may generate multiple shortened derivatives with enhanced antimicrobial activity but with significantly reduced proinflammatory activity. The aim of this study was to investigate cytokine release from oral keratinocyte and fibroblast cells in response to AM and eight fragments of the parent molecule, that were previously characterised in terms of their antimicrobial activities [3].

## 2. MATERIALS AND METHODS

### 2.1. Cell lines

Cell lines used were FIB originally derived from adult oral gingival keratinocytes, and human gingival fibroblasts (HGF) derived from the oral mucosa [9]. Dulbecco's Modified Eagle's Medium (DMEM), containing 10% foetal calf serum and penicillin/streptomycin, was used to culture cells. Cells were seeded into 96-well microtitre plates and when 90% confluent, were rendered quiescent by placing them in serum-starved medium for 24 hours prior to peptide exposure.

### 2.2. Peptides

Synthetic AM and AM fragments (residues 1-12, 1-21, 13-52, 16-21, 16-52, 22-52, 26-52, and 34-52) were purchased from Phoenix Pharmaceuticals (Karlsruhe, Germany).

AM fragment positions in the AM molecule are shown as follows:

**YRQSMNNFQGLR^12^S^13^FGC^16^RFGTC^21^T^22^VQKL^26^AH QIYQFT^34^DKDKDNVAPRSKISPQGY^52^**

### 2.3. Dose and time response experiments

AM and its fragments diluted in PBS to provide concentrations of 10^-6^, 10^-7^, 10^-8^, and 10^-9^ g/mL were used to determine the effect of concentration on cytokine response to these peptides. Cells were exposed to the four concentrations and supernatants were collected after 24 hours. Untreated cells were used as negative controls (cells incubated without AM or fragments). ELISAs (R *&* D Systems) were performed to measure interleukin 1-*α* (IL-1*α*), interleukin 1-*β* (IL-*β*), interleukin-6 (IL-6), and interleukin-8 (IL-8) levels.

AM and its fragments diluted in PBS at a concentration of 10^-9^ g/mL were used for time response experiments. Cells were exposed to AM and fragments, and supernatants collected at 1, 2, 4, 8 and 24 hours. ELISAs were then performed to measure IL-1*α*, IL-*β*, IL-6, and IL-8 levels. Untreated cells were used as negative controls.

### 2.4. MTT cell proliferation assay

Cells were plated in 96-well flat-bottomed plates in 200 *μ*L of medium and incubated overnight. Cells were serum-starved for 24 hours and then incubated with AM or fragments at a concentration of 10^-6^ g/mL. After 24 hours, 100 *μ*L of fresh medium was added into each well, together with 100 *μ*L of 5 mg/mL of MTT, and incubated for 2 hours. Media were then aspirated and 200 *μ*L of DMSO was added for 15 minutes. Plates were then read in a spectrophotometer at 595 nm.

### 2.5. Statistics

Results were analysed using two-way ANOVA and Bonferroni post tests (Prism 4 software).

## 3. RESULTS

### 3.1. MTT cell proliferation assay

Using the MTT assay with HGF cells, it was shown that treatment with AM or its fragments resulted in no significant decrease in viability or stimulation of the cells. Likewise, with FIB cells, treatment with AM or its fragments demonstrated no significant effect.

### 3.2. Dose response assay

The effect of AM and its fragments on cytokine production was shown not to be significantly dose dependent over the physiological range 10^-6^ to 10^-9^ g/mL. A final concentration of 10^-9^ g/mL was selected for further experiments.

### 3.3. Cytokine response of gingival fibroblasts

Cells were exposed to AM and individual fragments at a concentration of 10^-9^ g/mL. No significant increase of either IL-1*α* or IL-1*β* was observed over 24 hours or between the responses elicited by the whole molecule and its fragments (*<* 3 pg/mL in all cases). IL-6 and IL-8 were produced by the cells; however levels did not change significantly with either time or peptide tested (all levels *<* 140 pg/mL).

### 3.4. Cytokine response of gingival keratinocytes

**Figure 1:**
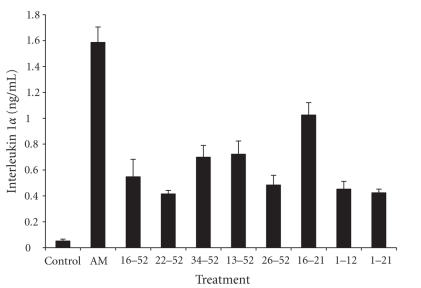
IL-1*α* response of FIB cells after a 24-hour exposure to AM and fragments (mean ± SD; n = 6). AM and individual fragments were each added to cultured cells at a final concentration of 10^-9^ g/mL. Antimicrobial fragments (16-52, 22-52, 34-52, and 13-52). Nonantimicrobial fragments (26-52, 16-21, 1-12, and 1-21).

**Figure 2:**
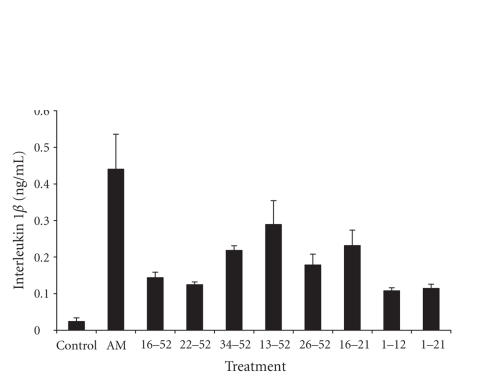
IL-1*β* response of FIB cells after a 24-hour exposure to AM and fragments (mean ± SD; n = 6). AM and individual fragments were each added to cultured cells at a final concentration of 10^-9^ g/mL. Antimicrobial fragments (16-52, 22-52, 34-52, and 13-52). Nonantimicrobial fragments (26-52, 16-21, 1-12, and 1-21).

Cells were exposed to AM and individual fragments at a concentration of 10^-9^ g/mL. A time-dependent increase in the release of IL-1*α* and IL-1*β* with exposure to AM and eight shortened derivatives was observed. Treatment with AM resulted in a significant increase in IL-1*α* and IL-1*β* at 4 hours (P *<* .001 and P *<* .05, resp.), 8 hours (P *<* .001), and 24 hours (P *<* .001) in comparison to controls. Treatment with all fragments resulted in a significant increase in both IL-1*α* and IL-1*β* at 24 hours (P *<* .001) in comparison to controls. Significant increases were also observed at 8 hours with the exception of the IL-1*α* response to fragment 1-12 and the IL-1*β* responses to fragments 1-12, 1-21, and 22-52. All fragments showed a significantly decreased response for both cytokines in comparison to the whole molecule at both 8 hours, with the exception of the IL-1*α* response to fragment 1-12, and 24 hours (60% overall mean reduction, P *<* .001). Responses at 24 hours to AM and individual peptides are shown in Figures 1 and 2. No consistent differences were demonstrated between the cytokine response elicited by the antimicrobial and nonantimicrobial fragments. IL-6 and IL-8 were produced by the cells; however, levels did not change significantly with time or peptide tested (all levels *<* 140 pg/mL).

## 4. DISCUSSION

Keratinocytes isolated from the skin synthesize and release the inflammatory cytokines IL-1, IL-6, and IL-8 in response to LPS, bacterial products, and other inflammatory cytokines [10]. In contrast, IL-1 is found in the intracellular compartment of fibroblasts but is not actively secreted [11]. With respect to the induction of cytokine production by AM stimulation, IL-6 has been shown to be produced from skin keratinocytes [12] and from Swiss 3T3 mouse fibroblast cells [13]. AM was found to stimulate basal secretion of IL-6 5.5-fold from Swiss 3T3 cells, while other peptides including AM (22-52) had much weaker stimulatory effects. The cytokine response of the oral keratinocytes and fibroblasts to AM in this study supports these findings.

AM binds to both specific AM and CGRP receptors and these are often expressed together in cells. The use of currently available inhibitors can help to define which receptor is actually mediating an effect. In terms of peptide-receptor interaction and activation of specific signal transduction pathways with fibroblasts cells, the effect of AM on IL-6 secretion from Swiss 3T3 cells has been shown to be inhibited by the AM-receptor antagonist AM (22-52) and a cAMP-dependent protein kinase inhibitor [13]. IL-1*α* and IL-6 release from oral keratinocytes, in response to AM, has been shown to be inhibited by AM (22-52) but not by the CGRP (8-37) antagonist (Hagi-Pavli, unpublished observations). It is known that the C-terminal section of the AM molecule binds to the receptor with greatest affinity [1]. In the current study, the C-terminal fragments generally elicited a higher IL-1*α* and IL-1*β* response than the N-terminal fragments. Further studies, to fully determine the receptor binding characteristics and signal transduction pathway(s) activated by shortened AM derivatives, are required.

Recently, it has been shown that the cationic antimicrobial peptide cathelicidin LL-37, found on the skin surface, is shortened by a serine protease-dependant mechanism into novel antimicrobial peptides with enhanced antimicrobial action but reduced proinflammatory activity [7]. It is suggested that, from the single human cathelicidin gene, multiple products are potentially generated with a range of biological activities, each relevant to the local environment in which they are released. The degradation of AM by host plasma membrane enzymes to major degradation products of 2-52 and 8-52, with smaller amounts of 26-52, 27-52, 28-52, and 33-52 [14], has been demonstrated. In a similar study [15], degradation of AM by matrix metalloproteinase-2 to fragments of 8-52, 11-52, 23-52, 29-52, 11-28, and 11-22 has also been shown. Thus postsecretory processing may generate up to 12 different shortened derivatives of AM as shown in these in vitro studies. The current study investigated the cytokine response to eight fragments that have been previously characterised in terms of their antimicrobial and vascular activities [3]. Further studies with other AM fragments that could possibly be generated in vivo, to further examine a possible relationship between antimicrobial and inflammatory activities, are warranted.

This study showed a clear difference in the potential immunomodulatory responses when oral keratinocytes were exposed to either AM or shortened derivatives of the parent molecule. These observations support previous studies in several cell, animal and human, systems that AM has both antimicrobial properties and also acts as a host stimulatory molecule. Peptide secretion followed by its processing could enable the epithelia to further modify the spectra of biological activity and regulate the balance between host immune modulation and inhibition of microbial growth.
